# Analytical and preparative applications of magnetic split-flow thin fractionation on several ion-labeled red blood cells

**DOI:** 10.1186/1477-044X-4-6

**Published:** 2006-12-19

**Authors:** Hweiyan Tsai, Ying S Fang, C Bor Fuh

**Affiliations:** 1 School of Applied Chemistry, Chung Shan Medical University, 110, Sec 1, Chien-Kuo N. Road, Taichung 402, Taiwan; 2 Department of Applied Chemistry, National Chi Nan University, 1, University Road, Puli, Nantou 545, Taiwan

## Abstract

**Background:**

Magnetic Split-flow thin (SPLITT) fractionation is a newly developed technique for separating magnetically susceptible particles. Particles with different field-induced velocities can be separated into two fractions by adjusting applied magnetic forces and flow-rates at inlets and outlets.

**Methods:**

Magnetic particles, Dynabeads, were used to test this new approach of field-induced velocity for susceptibility determination using magnetic SF at different magnetic field intensities. Reference measurements of magnetic susceptibility were made using a superconducting quantum interference device (SQUID) magnetometer. Various ion-labeled red blood cells (RBC) were used to study susceptibility determination and throughput parameters for analytical and preparative applications of magnetic SPLITT fractionation (SF), respectively. Throughputs were studied at different sample concentrations, magnetic field intensities, and channel flow-rates.

**Results:**

The susceptibilities of Dynabeads determined by SPLITT fractionation (SF) were consistent with those of reference measurement using a superconducting quantum interference device (SQUID) magnetometer. Determined susceptibilities of ion-labeled RBC were consistent within 9.6% variations at two magnetic intensities and different flow-rates. The determined susceptibilities differed by 10% from referenced measurements. The minimum difference in magnetic susceptibility required for complete separation was about 5.0 × 10^-6 ^[cgs]. Sample recoveries were higher than 92%. The throughput of magnetic SF was approximately 1.8 g/h using our experimental setup.

**Conclusion:**

Magnetic SF can provide simple and economical determination of particle susceptibility. This technique also has great potential for cell separation and related analysis. Continuous separations of ion-labeled RBC using magnetic SF were successful over 4 hours. The throughput was increased by 18 folds versus early study. Sample recoveries were 93.1 ± 1.8% in triplicate experiments.

## Background

Split-flow thin (SPLITT) fractionation has become useful separation techniques for macromolecules, colloids, and particles [1-14]. In SPLITT fractionation (SF), thin (< 0.5 mm) channels without packing stationary phase are used and different forces are applied perpendicularly to the channel flow for separations. SF and field-flow fractionation (FFF) are close family of separation techniques for macromolecules, colloids, and particles [15-18]. SF is mainly used for preparative applications whereas FFF is mainly used for analytical applications. Magnetic separation is fast, simple, and selective. Magnetic SF is a new member of SF family for separating magnetically susceptible colloids, and particles [2,3,5].

**Figure 1:**
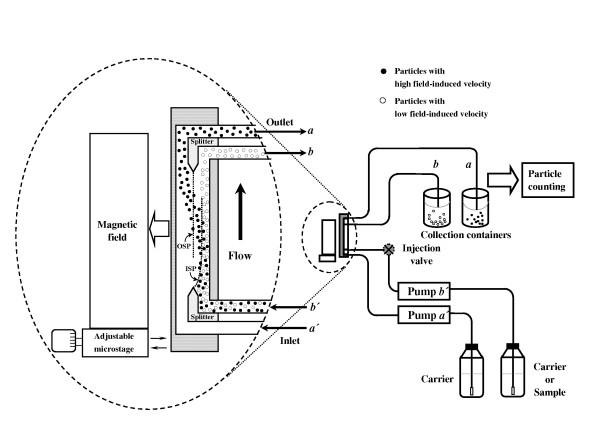
General schematic of magnetic SPLITT fractionation system with gravitational force parallel to the flow axis.

Typical magnetic SF has two inlets and two outlets, as shown in Fig. 1[5]. The parallel set up of separation channel and gravitational force can avoid gravity effect from separation. Samples in the carrier are introduced into one inlet and carriers were introduced into the other inlet with a higher flow-rate to confine samples in a small initial zone for better separation. The boundary planes between the two inlet and outlet flows are called the inlet splitting plane (ISP) and the outlet splitting plane (OSP), respectively. The positions of ISP and OSP are determined by the relative flow-rates of the two inlet and outlet substreams, respectively. Samples with low field-induced velocity are slightly affected by the magnetic force, thus move along the separation channel (not crossing the OSP) and exit at outlet *b*. Samples with higher field-induced velocity move toward the magnetic field direction (crossing the OSP) due to the magnetic force and exit at outlet *a *as they pass along the separation channel. Thus, samples are separated into different outlets based on their differences of field-induced velocity. The field-induced velocity mainly depends on sample magnetic susceptibility, sample diameter, carrier viscosity, and magnetic field intensity.

SF can be used for analytical and preparative applications using different applied forces [7,9,11]. In analytical applications, pulsed sample injection is used [5,7,9,11]. In preparative applications, samples are continuously pumped into one inlet and are fractionated into two outlets for collection and further identification. Magnetic SF has great potential for cell separations. Magnetic SF was used without combining gravitational force for separation of various ion-labeled RBC in this study. Various ion-labeled RBC were used to study susceptibility determination in analytical application and throughput parameters in preparative application using magnetic SF.

The theory of magnetic SF on fractional retrieval at outlets has been described in previous publications [3,5]. Therefore, we only briefly summarize it. To achieve complete separation (retrieval equaled 1 or 0) of samples with high (U_mh_) and low (U_ml_) magnetically induced velocities, the following equations must both be satisfied:

bLU_mh _≥ V˙
 MathType@MTEF@5@5@+=feaafiart1ev1aaatCvAUfKttLearuWrP9MDH5MBPbIqV92AaeXatLxBI9gBaebbnrfifHhDYfgasaacH8akY=wiFfYdH8Gipec8Eeeu0xXdbba9frFj0=OqFfea0dXdd9vqai=hGuQ8kuc9pgc9s8qqaq=dirpe0xb9q8qiLsFr0=vr0=vr0dc8meaabaqaciaacaGaaeqabaqabeGadaaakeaacuWGwbGvgaGaaaaa@2DEA@ (*b*), Fa = 1, and     (1)

bLU_ml _<V˙
 MathType@MTEF@5@5@+=feaafiart1ev1aaatCvAUfKttLearuWrP9MDH5MBPbIqV92AaeXatLxBI9gBaebbnrfifHhDYfgasaacH8akY=wiFfYdH8Gipec8Eeeu0xXdbba9frFj0=OqFfea0dXdd9vqai=hGuQ8kuc9pgc9s8qqaq=dirpe0xb9q8qiLsFr0=vr0=vr0dc8meaabaqaciaacaGaaeqabaqabeGadaaakeaacuWGwbGvgaGaaaaa@2DEA@ (*b*) - V˙
 MathType@MTEF@5@5@+=feaafiart1ev1aaatCvAUfKttLearuWrP9MDH5MBPbIqV92AaeXatLxBI9gBaebbnrfifHhDYfgasaacH8akY=wiFfYdH8Gipec8Eeeu0xXdbba9frFj0=OqFfea0dXdd9vqai=hGuQ8kuc9pgc9s8qqaq=dirpe0xb9q8qiLsFr0=vr0=vr0dc8meaabaqaciaacaGaaeqabaqabeGadaaakeaacuWGwbGvgaGaaaaa@2DEA@ (*b'*), Fa = 0     (2)

where Fa is the fraction of samples exiting at outlet *a *(fractional retrieval), b is the channel breadth, L is the channel length, V˙
 MathType@MTEF@5@5@+=feaafiart1ev1aaatCvAUfKttLearuWrP9MDH5MBPbIqV92AaeXatLxBI9gBaebbnrfifHhDYfgasaacH8akY=wiFfYdH8Gipec8Eeeu0xXdbba9frFj0=OqFfea0dXdd9vqai=hGuQ8kuc9pgc9s8qqaq=dirpe0xb9q8qiLsFr0=vr0=vr0dc8meaabaqaciaacaGaaeqabaqabeGadaaakeaacuWGwbGvgaGaaaaa@2DEA@ (*b*) and V˙
 MathType@MTEF@5@5@+=feaafiart1ev1aaatCvAUfKttLearuWrP9MDH5MBPbIqV92AaeXatLxBI9gBaebbnrfifHhDYfgasaacH8akY=wiFfYdH8Gipec8Eeeu0xXdbba9frFj0=OqFfea0dXdd9vqai=hGuQ8kuc9pgc9s8qqaq=dirpe0xb9q8qiLsFr0=vr0=vr0dc8meaabaqaciaacaGaaeqabaqabeGadaaakeaacuWGwbGvgaGaaaaa@2DEA@ (*b'*) are the volumetric flow-rates at outlet *b *and inlet *b'*, respectively. Complete separation is preferable for preparative application.

For samples with magnetically-induced velocities (U_m_) between U_mh _and U_ml_, i.e., V˙
 MathType@MTEF@5@5@+=feaafiart1ev1aaatCvAUfKttLearuWrP9MDH5MBPbIqV92AaeXatLxBI9gBaebbnrfifHhDYfgasaacH8akY=wiFfYdH8Gipec8Eeeu0xXdbba9frFj0=OqFfea0dXdd9vqai=hGuQ8kuc9pgc9s8qqaq=dirpe0xb9q8qiLsFr0=vr0=vr0dc8meaabaqaciaacaGaaeqabaqabeGadaaakeaacuWGwbGvgaGaaaaa@2DEA@ (*b*) - V˙
 MathType@MTEF@5@5@+=feaafiart1ev1aaatCvAUfKttLearuWrP9MDH5MBPbIqV92AaeXatLxBI9gBaebbnrfifHhDYfgasaacH8akY=wiFfYdH8Gipec8Eeeu0xXdbba9frFj0=OqFfea0dXdd9vqai=hGuQ8kuc9pgc9s8qqaq=dirpe0xb9q8qiLsFr0=vr0=vr0dc8meaabaqaciaacaGaaeqabaqabeGadaaakeaacuWGwbGvgaGaaaaa@2DEA@ (*b'*) ≤ bLU_m _<V˙
 MathType@MTEF@5@5@+=feaafiart1ev1aaatCvAUfKttLearuWrP9MDH5MBPbIqV92AaeXatLxBI9gBaebbnrfifHhDYfgasaacH8akY=wiFfYdH8Gipec8Eeeu0xXdbba9frFj0=OqFfea0dXdd9vqai=hGuQ8kuc9pgc9s8qqaq=dirpe0xb9q8qiLsFr0=vr0=vr0dc8meaabaqaciaacaGaaeqabaqabeGadaaakeaacuWGwbGvgaGaaaaa@2DEA@ (*b*), sample retrieval Fa can be calculated using:

Fa=bLUm−V˙(b)+V˙(b′)V˙(b′)     (3)
 MathType@MTEF@5@5@+=feaafiart1ev1aaatCvAUfKttLearuWrP9MDH5MBPbIqV92AaeXatLxBI9gBaebbnrfifHhDYfgasaacH8akY=wiFfYdH8Gipec8Eeeu0xXdbba9frFj0=OqFfea0dXdd9vqai=hGuQ8kuc9pgc9s8qqaq=dirpe0xb9q8qiLsFr0=vr0=vr0dc8meaabaqaciaacaGaaeqabaqabeGadaaakeaacqqGgbGrcqqGHbqycqGH9aqpdaWcaaqaaiabbkgaIjabbYeamjabbwfavnaaBaaaleaacqqGTbqBaeqaaOGaeyOeI0IafmOvayLbaiaacqGGOaakcqWGIbGycqGGPaqkcqGHRaWkcuWGwbGvgaGaaiabcIcaOiqbdkgaIzaafaGaeiykaKcabaGafmOvayLbaiaacqGGOaakcuWGIbGygaqbaiabcMcaPaaacaWLjaGaaCzcamaabmaabaGaeG4mamdacaGLOaGaayzkaaaaaa@47AF@

U_m _can be calculated from the balance of magnetic force (0.5 ΔχV∇*B*^2^) and drag force (3πη*d *U_m_) using:

Um=d236ηΔχ∇B2     (4)
 MathType@MTEF@5@5@+=feaafiart1ev1aaatCvAUfKttLearuWrP9MDH5MBPbIqV92AaeXatLxBI9gBaebbnrfifHhDYfgasaacH8akY=wiFfYdH8Gipec8Eeeu0xXdbba9frFj0=OqFfea0dXdd9vqai=hGuQ8kuc9pgc9s8qqaq=dirpe0xb9q8qiLsFr0=vr0=vr0dc8meaabaqaciaacaGaaeqabaqabeGadaaakeaacqWGvbqvdaWgaaWcbaGaemyBa0gabeaakiabg2da9maalaaabaGaemizaq2aaWbaaSqabeaacqaIYaGmaaaakeaacqaIZaWmcqaI2aGniiGacqWF3oaAaaGaeuiLdqKae83XdmMaey4bIeTaemOqai0aaWbaaSqabeaacqaIYaGmaaGccaWLjaGaaCzcamaabmaabaGaeGinaqdacaGLOaGaayzkaaaaaa@4140@

where Δχ = χ_p _- χ_c_, χ_p _and χ_c _are the respective magnetic susceptibilities of particles and carriers, V is the volume of particle, η is the fluid viscosity, *d *is the effective diameter of spherical particle, ∇ is the gradient operator, and *B *is the magnetic field intensity.

Magnetic susceptibility is an important parameter for magnetic separation. Retrieval between 0 and 1 is preferable for magnetic susceptibility determination. Magnetic susceptibility of ion-labeled RBC can be deduced from combining equations (3) and (4) with simple mathematic treatments.

Δχ=36η[FaV˙(b′)+V˙(b)−V˙(b′)]bLd2∇B2     (5)
 MathType@MTEF@5@5@+=feaafiart1ev1aaatCvAUfKttLearuWrP9MDH5MBPbIqV92AaeXatLxBI9gBaebbnrfifHhDYfgasaacH8akY=wiFfYdH8Gipec8Eeeu0xXdbba9frFj0=OqFfea0dXdd9vqai=hGuQ8kuc9pgc9s8qqaq=dirpe0xb9q8qiLsFr0=vr0=vr0dc8meaabaqaciaacaGaaeqabaqabeGadaaakeaacqqHuoariiGacqWFhpWycqGH9aqpdaWcaaqaaiabiodaZiabiAda2iab=D7aOjabcUfaBjabdAeagjabdggaHjqbdAfawzaacaGaeiikaGIafmOyaiMbauaacqGGPaqkcqGHRaWkcuWGwbGvgaGaaiabcIcaOiabdkgaIjabcMcaPiabgkHiTiqbdAfawzaacaGaeiikaGIafmOyaiMbauaacqGGPaqkcqGGDbqxaeaacqqGIbGycqqGmbatcqWGKbazdaahaaWcbeqaaiabikdaYaaakiabgEGirlabdkeacnaaCaaaleqabaGaeGOmaidaaaaakiaaxMaacaWLjaWaaeWaaeaacqaI1aqnaiaawIcacaGLPaaaaaa@545E@

Magnetic susceptibility of ion-labeled RBC can be calculated from known carrier susceptibility and experimental parameters [η, b, L, ∇B^2^, d, *Fa*, V˙
 MathType@MTEF@5@5@+=feaafiart1ev1aaatCvAUfKttLearuWrP9MDH5MBPbIqV92AaeXatLxBI9gBaebbnrfifHhDYfgasaacH8akY=wiFfYdH8Gipec8Eeeu0xXdbba9frFj0=OqFfea0dXdd9vqai=hGuQ8kuc9pgc9s8qqaq=dirpe0xb9q8qiLsFr0=vr0=vr0dc8meaabaqaciaacaGaaeqabaqabeGadaaakeaacuWGwbGvgaGaaaaa@2DEA@ (*b*), and V˙
 MathType@MTEF@5@5@+=feaafiart1ev1aaatCvAUfKttLearuWrP9MDH5MBPbIqV92AaeXatLxBI9gBaebbnrfifHhDYfgasaacH8akY=wiFfYdH8Gipec8Eeeu0xXdbba9frFj0=OqFfea0dXdd9vqai=hGuQ8kuc9pgc9s8qqaq=dirpe0xb9q8qiLsFr0=vr0=vr0dc8meaabaqaciaacaGaaeqabaqabeGadaaakeaacuWGwbGvgaGaaaaa@2DEA@ (*b'*)].

## Methods

The assembly of separation channels was the same as previous work [3]. Channel was assembled using three layers of cut-out Mylar sandwiched between two plastic sheets. Plastic screws were used beside the splitter edge to seal the Mylar layers and avoid carrier leakage at high flow-rates. Four sets of channel length and channel breadth [(70 mm, 5 mm), (140 mm, 5 mm), (70 mm, 10 mm), and (140 mm, 10 mm)] were used. All experiments of susceptibility determinations used 70 mm of channel length, 5 mm and 10 mm of channel breadths. All channels used 0.25 mm of thickness.

Magnetic fields were generated by assembling one pair of rare-earth magnets [neodymium-iron-boron (Nd-Fe-B)] and soft ion pole pieces, which conducted the magnetic fluxes to the interpolar gap. The maximum energy products of the Nd-Fe-B magnets were 3.5 × 10^7^G-Oe. Magnetic field intensities were generated from 5 mm and 10 mm of gap widths with 70 mm and 140 mm of gap lengths. Magnetic field was measured using a Gaussmeter and a Hall-effect probe (Model 5080, F. W. Bell, Orlando, FL, USA). The saturated magnetic field intensities (Bo) were 1.20 Tesla and 0.72 Tesla for 5 mm and 10 mm of gapwidths, respectively. Microstages were used to adjust magnetic field intensities.

The carrier of study was phosphate buffered saline (PBS) with pH of 7.02 and viscosity of 1.0 × 10^-2 ^g cm^-1^s^-1^. Samples and carriers were delivered into the separation channel using a microtubing pump (Eyela Mp-3, Rikakikai, Tokyo, Japan) and LC pump (SSI series II, State College, PA, USA), respectively. Sample verification was done using light microscopy (Olympus BX-50, Tokyo, Japan). Sample counting was done using a hemacytometer (cell-counting chamber with microscalar grids having fixed volume). Trypan blue, manganese sulfate and iron nitrate were purchased from Sigma Chemical (St. Louis, MO, USA). Copper chloride was purchased from the Aldrich Chemicals (St. Louis, MO, USA). Erbium chloride was purchased from Strem (Newburyport, MA, USA). Dynabeads M-450 (4.5 μm) were uniform particles doped with iron oxides. Yeasts were purchased from local bakery of Taichung. Blood cells were obtained from the Jen-Ai Hospital in Dali (Taichung County, Taiwan).

Fresh blood from hospital was centrifuged at 50 g for 5 min to remove plasma. The cells were then washed with PBS and centrifuged three times before labeling. Various concentrations of labeling ion were obtained by diluting 100 mM of prepared stock solutions. Various ion-labeled RBC were prepared by mixing 1 mL of labeling ions at fixed concentrations with 9 mL of solutions containing 4.0 × 10^5 ^RBC and incubating in ice for 30 min with shaking every 10 min. All ion-labeled cells were washed three times with PBS solution before use to remove unlabeled ions. The labeling ions were Er^3+^, Fe^3+^, Mn^2+^, and Cu^2+^. Dye exclusion testing was carried out using trypan blue stain and a hemacytometer. This method was based on the assumption that viable cells did not take up dyes, whereas nonviable cells did. For viability testing, 0.5 mL containing 1.0 × 10^6^cell suspensions were mixed thoroughly with 0.5 mL of 0.4% (w/v) trypan blue solution for 5 min before counting.

Fractional retrieval of samples at outlet a (Fa) was calculated using the following equation:

Fa=NaNa+Nb     (5)
 MathType@MTEF@5@5@+=feaafiart1ev1aaatCvAUfKttLearuWrP9MDH5MBPbIqV92AaeXatLxBI9gBaebbnrfifHhDYfgasaacH8akY=wiFfYdH8Gipec8Eeeu0xXdbba9frFj0=OqFfea0dXdd9vqai=hGuQ8kuc9pgc9s8qqaq=dirpe0xb9q8qiLsFr0=vr0=vr0dc8meaabaqaciaacaGaaeqabaqabeGadaaakeaacqqGgbGrcqqGHbqycqGH9aqpdaWcaaqaaiabb6eaojabbggaHbqaaiabb6eaojabbggaHjabgUcaRiabb6eaojabbkgaIbaacaWLjaGaaCzcamaabmaabaGaeGynaudacaGLOaGaayzkaaaaaa@3C0B@

where Na and Nb were the number of particles exiting at outlets *a *and *b*, respectively. The sum of Fa and Fb was equal to one. The recovery percentage of RBC was calculated by adding the total number of RBC exiting at both outlets and dividing by the total number of RBC entering at the inlets. A minimum of 200 cells was counted in each retrieval experiment.

Pulsed sample injections were used for susceptibility determination in analytical application and optimization of separation conditions for continuous particle separation. The loop volume of pulsed sample injection was 0.7 ml. Reference measurements of magnetic susceptibility were made using an MPMS5 model superconducting quantum interference device (SQUID) magnetometer from Quantum Design (San Diego, CA, USA). Magnetic field intensities from 1.0 × 10^4 ^to 2.0 × 10^4 ^gauss were used for susceptibility measurements in SQUID. The cgs system and volume magnetic susceptibility, χ, are used throughout this study for convenient calculation unless otherwise indicated.

In preparative application, samples were continuously introduced into one inlet and fractionated RBC and yeasts were collected at two outlets. Fractionated RBC and yeasts were verified using microscopy for size and shape, and verified by permanent magnets for susceptibility at the end of each hour run during continuous separation.

## Results and discussion

### The determination of Dynabeads susceptibility

Magnetic susceptibility determination of particles using analytical SF was reported in the literature [5]. The susceptibility determination was based on the calculation of fractional retrieval of particle under controlled flow-rates and magnetic fields. We tried a new approach of magnetically-induced velocity (U_m_) for susceptibility determination of ion-labeled RBC in this study. The new approach of field-induced velocity (U_m_) was derived using the balance of magnetic force (0.5 ΔχV∇*B*^2^) and drag force (3πη*d *U_m_). Susceptibility determination was calculated from the known experimental parameters of carrier viscosity, channel dimension, fractional retrieval, particle diameter, magnetic intensity, and flow-rates of inlet and outlet, as shown in equation 5. Magnetic particles, Dynabeads, were used to test this new approach of field-induced velocity for susceptibility determination using magnetic SF at different magnetic field intensities, as shown in Table 1. Total flow-rates can be used up to 6 mL/min for high susceptibility particles like Dynabeads. The susceptibilities of Dynabeads were determined at different magnetic intensities and total flow-rates. Determined susceptibilities of Dynabeads were consistent with relative standard deviation (RSD) within 5% variations and differed by a 5% range from reference measurements using SQUID for all measured conditions. The results indicated that magnetic SF could provide simple and economical determination of particle susceptibility.

**Table 1:** Determined magnetic susceptibility of Dynabeads using magnetic SF

**Interpolar gapwidth** ** (mm) **	**Inlet flow-rate ** ** (ml min^-1^) **		**Outlet flow-rate ** ** (ml min^-1^) **		**Fa (%) ** ** (n = 5) **	Δ χ¯ MathType@MTEF@5@5@+=feaafiart1ev1aaatCvAUfKttLearuWrP9MDH5MBPbIqV92AaeXatLxBI9gBaebbnrfifHhDYfgasaacH8akY=wiFfYdH8Gipec8Eeeu0xXdbba9frFj0=OqFfea0dXdd9vqai=hGuQ8kuc9pgc9s8qqaq=dirpe0xb9q8qiLsFr0=vr0=vr0dc8meaabaqaciaacaGaaeqabaqabeGadaaakeaaiiGacuWFhpWygaqeaaaa@2E82@**± SD (dimensionless, cgs) (10^-6^) ** ** (n = 5) **	**RSD** ** (%) **
				
	V˙ MathType@MTEF@5@5@+=feaafiart1ev1aaatCvAUfKttLearuWrP9MDH5MBPbIqV92AaeXatLxBI9gBaebbnrfifHhDYfgasaacH8akY=wiFfYdH8Gipec8Eeeu0xXdbba9frFj0=OqFfea0dXdd9vqai=hGuQ8kuc9pgc9s8qqaq=dirpe0xb9q8qiLsFr0=vr0=vr0dc8meaabaqaciaacaGaaeqabaqabeGadaaakeaaieqacuWFwbGvgaGaaaaa@2DF0@**(a')**	V˙ MathType@MTEF@5@5@+=feaafiart1ev1aaatCvAUfKttLearuWrP9MDH5MBPbIqV92AaeXatLxBI9gBaebbnrfifHhDYfgasaacH8akY=wiFfYdH8Gipec8Eeeu0xXdbba9frFj0=OqFfea0dXdd9vqai=hGuQ8kuc9pgc9s8qqaq=dirpe0xb9q8qiLsFr0=vr0=vr0dc8meaabaqaciaacaGaaeqabaqabeGadaaakeaaieqacuWFwbGvgaGaaaaa@2DF0@**(b')**	V˙ MathType@MTEF@5@5@+=feaafiart1ev1aaatCvAUfKttLearuWrP9MDH5MBPbIqV92AaeXatLxBI9gBaebbnrfifHhDYfgasaacH8akY=wiFfYdH8Gipec8Eeeu0xXdbba9frFj0=OqFfea0dXdd9vqai=hGuQ8kuc9pgc9s8qqaq=dirpe0xb9q8qiLsFr0=vr0=vr0dc8meaabaqaciaacaGaaeqabaqabeGadaaakeaaieqacuWFwbGvgaGaaaaa@2DF0@**(a)**	V˙ MathType@MTEF@5@5@+=feaafiart1ev1aaatCvAUfKttLearuWrP9MDH5MBPbIqV92AaeXatLxBI9gBaebbnrfifHhDYfgasaacH8akY=wiFfYdH8Gipec8Eeeu0xXdbba9frFj0=OqFfea0dXdd9vqai=hGuQ8kuc9pgc9s8qqaq=dirpe0xb9q8qiLsFr0=vr0=vr0dc8meaabaqaciaacaGaaeqabaqabeGadaaakeaaieqacuWFwbGvgaGaaaaa@2DF0@**(b)**			
5	^a^3.0	1.0	2.0	2.0	46 ± 1	20300 ± 1000	4.9
	^b^4.5	1.5	3.0	3.0	92 ± 1	21000 ± 900	4.3
10	^c^3.0	1.0	2.0	2.0	36 ± 1	20200 ± 940	4.6
	^d^4.5	1.5	3.0	3.0	78 ± 1	19800 ± 1000	5.0

∇
 MathType@MTEF@5@5@+=feaafiart1ev1aaatCvAUfKttLearuWrP9MDH5MBPbIqV92AaeXatLxBI9gBaebbnrfifHhDYfgasaacH8akY=wiFfYdH8Gipec8Eeeu0xXdbba9frFj0=OqFfea0dXdd9vqai=hGuQ8kuc9pgc9s8qqaq=dirpe0xb9q8qiLsFr0=vr0=vr0dc8meaabaqaciaacaGaaeqabaqabeGadaaakeaacqGHhis0aaa@2E32@ B^2 ^(G^2^/μm): a = 61, b = 116, c = 29, d = 57

Δ χ¯
 MathType@MTEF@5@5@+=feaafiart1ev1aaatCvAUfKttLearuWrP9MDH5MBPbIqV92AaeXatLxBI9gBaebbnrfifHhDYfgasaacH8akY=wiFfYdH8Gipec8Eeeu0xXdbba9frFj0=OqFfea0dXdd9vqai=hGuQ8kuc9pgc9s8qqaq=dirpe0xb9q8qiLsFr0=vr0=vr0dc8meaabaqaciaacaGaaeqabaqabeGadaaakeaaiiGacuWFhpWygaqeaaaa@2E82@ ± SD of Dynabeads from reference measurement using SQUID were (20000 ± 780) × (10^-6^) (n = 10)

### The determination of ion-labeled RBC susceptibility

**Figure 2:**
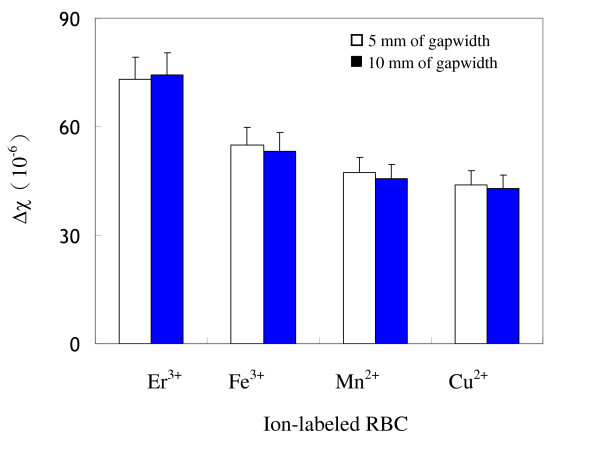
**Determined susceptibilities of various ion-labeled RBC at different interpolar gapwidths**. The concentration of labeled ions was 100 mM. The flow rate conditions were [V˙
 MathType@MTEF@5@5@+=feaafiart1ev1aaatCvAUfKttLearuWrP9MDH5MBPbIqV92AaeXatLxBI9gBaebbnrfifHhDYfgasaacH8akY=wiFfYdH8Gipec8Eeeu0xXdbba9frFj0=OqFfea0dXdd9vqai=hGuQ8kuc9pgc9s8qqaq=dirpe0xb9q8qiLsFr0=vr0=vr0dc8meaabaqaciaacaGaaeqabaqabeGadaaakeaacuWGwbGvgaGaaaaa@2DEA@ (mL/min): *a' *= 1.5, *b' *= 0.5, *a *= 1.2, *b *= 0.8]. Magnetic field conditions [∇B^2 ^(gauss^2^/μm)] and interpolar gapwidths (mm) were: (A) 6800 ± 50 at 5 mm, (B) 3600 ± 40 at 10 mm.

**Figure 3:**
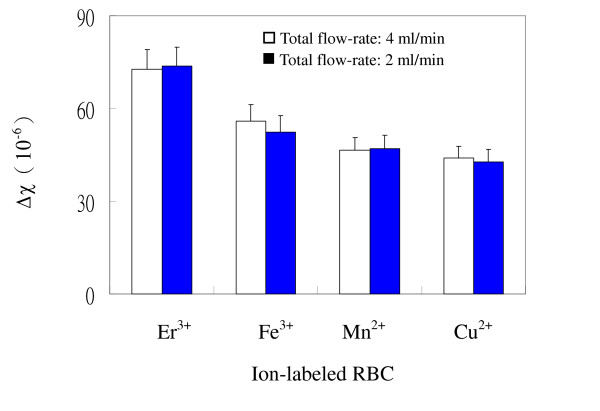
**Determined susceptibilities of various ion-labeled RBC at different flow-rates**. The concentration of labeled ions was 100 mM. The flow-rate conditions were: (A) [V˙
 MathType@MTEF@5@5@+=feaafiart1ev1aaatCvAUfKttLearuWrP9MDH5MBPbIqV92AaeXatLxBI9gBaebbnrfifHhDYfgasaacH8akY=wiFfYdH8Gipec8Eeeu0xXdbba9frFj0=OqFfea0dXdd9vqai=hGuQ8kuc9pgc9s8qqaq=dirpe0xb9q8qiLsFr0=vr0=vr0dc8meaabaqaciaacaGaaeqabaqabeGadaaakeaacuWGwbGvgaGaaaaa@2DEA@ (mL/min): *a' *= 1.5, *b' *= 0.5, *a *= 1.2, *b *= 0.8], (B) [V˙
 MathType@MTEF@5@5@+=feaafiart1ev1aaatCvAUfKttLearuWrP9MDH5MBPbIqV92AaeXatLxBI9gBaebbnrfifHhDYfgasaacH8akY=wiFfYdH8Gipec8Eeeu0xXdbba9frFj0=OqFfea0dXdd9vqai=hGuQ8kuc9pgc9s8qqaq=dirpe0xb9q8qiLsFr0=vr0=vr0dc8meaabaqaciaacaGaaeqabaqabeGadaaakeaacuWGwbGvgaGaaaaa@2DEA@ (mL/min): *a' *= 3.0, *b' *= 1.0, *a *= 2.4, *b *= 1.6]. Interpolar gapwidth was 5 mm. Magnetic field conditions [∇B^2 ^(gauss^2^/μm)] were: (A) 6330 ± 50, (B) 14600 ± 200.

Low susceptibility samples of ion-labeled RBC were studied for susceptibility determination using magnetic SF followed high susceptibility samples. Figure 2 shows the determined susceptibilities of several ion-labeled RBC at 2 mL/min of total flow-rate but different magnetic intensities with 5 mm and 10 mm of interpolar gapwidths. Determined susceptibilities of ion-labeled RBC were within 9.6% variations at two magnetic intensities for all of ion-labeled RBC. Figure 3 shows the determined susceptibilities of ion-labeled RBC at different flow-rates and magnetic intensities. Determined susceptibilities were within 9.8% variations for all ion-labeled RBC. Overall, determined susceptibilities of ion-labeled RBC were within 10% variations at various flow-rates and magnetic intensities.

### Separation of mixtures of yeasts and ion-labeled RBC

Separation of a specific component from a mixture using magnetic SF usually takes several steps to complete. The number of steps for separation of one component from the rest of mixture depends on the characteristics of targeted components and the mixture matrix. Preparative applications of magnetic SF for cells are very useful to cell purification and analysis. Cell mixtures of yeasts and ion-labeled RBC were used to demonstrate the feasibility of preparative application for magnetic SF.

Pulsed sample injection was used to optimize experimental conditions of continuous separation. The applied magnetic forces and the flow-rates of inlets and outlets are important parameters for applications of magnetic SF, as shown in equations 1–4. The retrieval calculation of equations 3 and 4 provided a useful guide for the first trial and followed modifications of experimental parameters to approach complete or continuous separation.

A 70 mm of channel length and 5 mm of channel breadth and interpolar gapwidth were used for this separation. The flow-rate conditions were [V˙
 MathType@MTEF@5@5@+=feaafiart1ev1aaatCvAUfKttLearuWrP9MDH5MBPbIqV92AaeXatLxBI9gBaebbnrfifHhDYfgasaacH8akY=wiFfYdH8Gipec8Eeeu0xXdbba9frFj0=OqFfea0dXdd9vqai=hGuQ8kuc9pgc9s8qqaq=dirpe0xb9q8qiLsFr0=vr0=vr0dc8meaabaqaciaacaGaaeqabaqabeGadaaakeaacuWGwbGvgaGaaaaa@2DEA@ (mL/min): *a' *= 1.5, *b' *= 0.5, *a *= 1.2, *b *= 0.8]. The magnetic field condition [∇B^2 ^(gauss^2^/μm)] needed for separation was found to be 13600, which was about 4% higher than the theoretical calculation. The experimental parameters of magnetic field intensity and flow-rate conditions used for separation were consistent with those predicted from equations 1–2 within 6% variations in terms of retrievals. Ion-labeled RBC were driven far enough by the magnetic force to cross the OSP and came out at outlet *a *under this separation conditions. Yeasts were less driven by the magnetic force and did not cross the OSP and came out at outlet *b*. For fixed flow-rate conditions, samples of high magnetic susceptibility required lower magnetic field intensity and samples of low magnetic susceptibility required higher magnetic field intensity for separation. The fractionated samples at outlets were collected and examined using microscopy for their sizes and shapes. Yeasts and ion-labeled RBC were injected separately and recovered fully at the respective outlet to ensure successful separation of the component mixture. Reinjection of collected fractions came out at the same outlet. The mixtures of yeasts and ion-labeled RBC were prepared in the same amounts as individual injections, and were injected into the magnetic SF system. The components of eluate at different outlets were examined by microscopy. All components of mixtures came out at the same outlets as their individual component injections. Sample recovery ranged from 91 to 96% with a mean of 93.5 ± 2.5%. There were about 4% retrieval variations. Experimental retrievals (Fa) differed by 10% from those theoretical prediction of equations 1–3. The mixture of ion-labeled RBC and yeasts were successfully fractionated based on the retrieval calculation and microscopic examination.

### Throughput of continuous separation

Throughput is a very important parameter for applications of continuous separation. The throughput of SF is proportional to sample concentration in the feed stream, channel cross section (channel length × channel breadth), volumetric flow-rate of the sample stream, and the applied field intensity [1]. These variables were studied to optimize the throughput of magnetic SF. The tested sample concentrations of feed stream were 0.1%, 0.25%, 0.5%, 1.0%, 1.1%, and 1.2% (w/v) for separation of yeasts and ion-labeled RBC using previous experimental conditions [V˙
 MathType@MTEF@5@5@+=feaafiart1ev1aaatCvAUfKttLearuWrP9MDH5MBPbIqV92AaeXatLxBI9gBaebbnrfifHhDYfgasaacH8akY=wiFfYdH8Gipec8Eeeu0xXdbba9frFj0=OqFfea0dXdd9vqai=hGuQ8kuc9pgc9s8qqaq=dirpe0xb9q8qiLsFr0=vr0=vr0dc8meaabaqaciaacaGaaeqabaqabeGadaaakeaacuWGwbGvgaGaaaaa@2DEA@ (mL/min): *a' *= 1.5, *b' *= 0.5, *a *= 1.2, *b *= 0.8 and ∇B^2 ^(gauss^2^/μm): 13600]. Mixing effects of separation might occur since calculated retrievals changed more than 20% for 1.1% (w/v) of sample concentration. The retrievals were within 5% variations for sample concentration ranged from 0.1% to 1.0%(w/v). The maximum feed concentration was around 1.0% (w/v). The throughputs of magnetic SF were studied using three channel cross-sections [channel length (mm) × channel breadth (mm): (70 × 5), (140 × 5), (140 × 10)]. The flow-rate conditions used to study the effect of channel cross-section on throughput were same as those used in the study of sample concentration. The throughput was increased to ~1.7 times when the channel length was varied from 70 mm to 140 mm at a fixed (5 mm) channel breadth. However, the throughput was only increased to ~1.2 times when the channel breadth was doubled at a fixed (140 mm) channel length. The difference of the throughput change was due to the sensitivity of the magnetic field intensity on the interpolar gapwidth, i.e., the channel breadth. Either the flow-rate or applied force needs to be adjusted for the changes of channel length and breadth to balance the throughput. The throughput can be increased about 20–70% using larger channel cross-sections by doubling the channel length or channel breadth.

Higher flow-rates of sample stream decreased particle residence time in the separation channel and thus required greater magnetic field intensities to achieve the same separation resolution at fixed flow ratios of inlet [V˙
 MathType@MTEF@5@5@+=feaafiart1ev1aaatCvAUfKttLearuWrP9MDH5MBPbIqV92AaeXatLxBI9gBaebbnrfifHhDYfgasaacH8akY=wiFfYdH8Gipec8Eeeu0xXdbba9frFj0=OqFfea0dXdd9vqai=hGuQ8kuc9pgc9s8qqaq=dirpe0xb9q8qiLsFr0=vr0=vr0dc8meaabaqaciaacaGaaeqabaqabeGadaaakeaacuWGwbGvgaGaaaaa@2DEA@ (*a'*)/V˙
 MathType@MTEF@5@5@+=feaafiart1ev1aaatCvAUfKttLearuWrP9MDH5MBPbIqV92AaeXatLxBI9gBaebbnrfifHhDYfgasaacH8akY=wiFfYdH8Gipec8Eeeu0xXdbba9frFj0=OqFfea0dXdd9vqai=hGuQ8kuc9pgc9s8qqaq=dirpe0xb9q8qiLsFr0=vr0=vr0dc8meaabaqaciaacaGaaeqabaqabeGadaaakeaacuWGwbGvgaGaaaaa@2DEA@ (*b'*)] and outlet [V˙
 MathType@MTEF@5@5@+=feaafiart1ev1aaatCvAUfKttLearuWrP9MDH5MBPbIqV92AaeXatLxBI9gBaebbnrfifHhDYfgasaacH8akY=wiFfYdH8Gipec8Eeeu0xXdbba9frFj0=OqFfea0dXdd9vqai=hGuQ8kuc9pgc9s8qqaq=dirpe0xb9q8qiLsFr0=vr0=vr0dc8meaabaqaciaacaGaaeqabaqabeGadaaakeaacuWGwbGvgaGaaaaa@2DEA@ (*a*)/V˙
 MathType@MTEF@5@5@+=feaafiart1ev1aaatCvAUfKttLearuWrP9MDH5MBPbIqV92AaeXatLxBI9gBaebbnrfifHhDYfgasaacH8akY=wiFfYdH8Gipec8Eeeu0xXdbba9frFj0=OqFfea0dXdd9vqai=hGuQ8kuc9pgc9s8qqaq=dirpe0xb9q8qiLsFr0=vr0=vr0dc8meaabaqaciaacaGaaeqabaqabeGadaaakeaacuWGwbGvgaGaaaaa@2DEA@ (*b*)]. The effect of sample flow-rates [V˙
 MathType@MTEF@5@5@+=feaafiart1ev1aaatCvAUfKttLearuWrP9MDH5MBPbIqV92AaeXatLxBI9gBaebbnrfifHhDYfgasaacH8akY=wiFfYdH8Gipec8Eeeu0xXdbba9frFj0=OqFfea0dXdd9vqai=hGuQ8kuc9pgc9s8qqaq=dirpe0xb9q8qiLsFr0=vr0=vr0dc8meaabaqaciaacaGaaeqabaqabeGadaaakeaacuWGwbGvgaGaaaaa@2DEA@ (*b'*)] on throughput was studied using two flow-rate conditions [V˙
 MathType@MTEF@5@5@+=feaafiart1ev1aaatCvAUfKttLearuWrP9MDH5MBPbIqV92AaeXatLxBI9gBaebbnrfifHhDYfgasaacH8akY=wiFfYdH8Gipec8Eeeu0xXdbba9frFj0=OqFfea0dXdd9vqai=hGuQ8kuc9pgc9s8qqaq=dirpe0xb9q8qiLsFr0=vr0=vr0dc8meaabaqaciaacaGaaeqabaqabeGadaaakeaacuWGwbGvgaGaaaaa@2DEA@ (mL/min): *a' *= 3.0, *b' *= 1.0, *a *= 2.4, *b *= 1.6 and *a' *= 4.5, *b' *= 1.5, *a *= 3.6, *b *= 2.4] at 140 mm of channel length and 5 mm of channel breadth. The needed ∇B^2 ^for 1.0 mL/min and 1.5 mL/min of sample flow-rates [V˙
 MathType@MTEF@5@5@+=feaafiart1ev1aaatCvAUfKttLearuWrP9MDH5MBPbIqV92AaeXatLxBI9gBaebbnrfifHhDYfgasaacH8akY=wiFfYdH8Gipec8Eeeu0xXdbba9frFj0=OqFfea0dXdd9vqai=hGuQ8kuc9pgc9s8qqaq=dirpe0xb9q8qiLsFr0=vr0=vr0dc8meaabaqaciaacaGaaeqabaqabeGadaaakeaacuWGwbGvgaGaaaaa@2DEA@ (*b'*)] were found to be 14600 gauss^2^/μm and 21300 gauss^2^/μm, respectively. Higher flow-rates of sample stream can be used for greater magnetic field intensities at the same separation resolution. Greater magnetic field intensity can increase the throughput. The averaged sample recoveries were 92.6 ± 1.8% and 93.5 ± 1.8% for 1.0 mL/min and 1.5 mL/min of sample flow-rates [V˙
 MathType@MTEF@5@5@+=feaafiart1ev1aaatCvAUfKttLearuWrP9MDH5MBPbIqV92AaeXatLxBI9gBaebbnrfifHhDYfgasaacH8akY=wiFfYdH8Gipec8Eeeu0xXdbba9frFj0=OqFfea0dXdd9vqai=hGuQ8kuc9pgc9s8qqaq=dirpe0xb9q8qiLsFr0=vr0=vr0dc8meaabaqaciaacaGaaeqabaqabeGadaaakeaacuWGwbGvgaGaaaaa@2DEA@ (*b'*)], respectively. The highest flow-rates of sample stream useful for separation was 3 mL/min using the present setup [V˙
 MathType@MTEF@5@5@+=feaafiart1ev1aaatCvAUfKttLearuWrP9MDH5MBPbIqV92AaeXatLxBI9gBaebbnrfifHhDYfgasaacH8akY=wiFfYdH8Gipec8Eeeu0xXdbba9frFj0=OqFfea0dXdd9vqai=hGuQ8kuc9pgc9s8qqaq=dirpe0xb9q8qiLsFr0=vr0=vr0dc8meaabaqaciaacaGaaeqabaqabeGadaaakeaacuWGwbGvgaGaaaaa@2DEA@ (mL/min): *a' *= 7.5, *b' *= 3.0, *a *= 6, *b *= 4.5 and ∇B^2^: 23100 gauss^2^/μm]. The throughput was 1.8 g/h using 1% (w/v) of sample concentration. Continuous separation of magnetic SF was successfully operated over 4 hours. The minimum difference of magnetic susceptibility required for complete separation was about 4.0 × 10^-6 ^[cgs], as determined from the known susceptibility of Er^3+^-labeled RBC.

## Conclusion

Magnetic SF can provide simple and economical determination of particle susceptibility. The susceptibilities determined by magnetic SF were consistent with those of reference measurements using a superconducting quantum interference device (SQUID) magnetometer. This technique also has great potential for cell separation and related analysis. Several parameters including applied magnetic forces, channel length, channel breadth, and sample flow-rates were optimized for the throughput. Continuous separations of ion-labeled RBC using magnetic SF were successfully operated over 4 hours. The throughput was increased by 18 folds versus early study [2]. The total averages of sample recoveries were 93.1 ± 1.8% in triplicate experiments. Using longer channel lengths, broader channel breadths, and stronger magnetic fields in the feature can scale up the throughput. Greater magnetic field intensity using a superconducting device would require for the further increase of throughput.

## Abbreviations

SPLITT: Split-flow thin.

SF: SPLITT fractionation.

RBC: red blood cells

SQUID: superconducting quantum interference device.

FFF: field-flow fractionation

ISP: inlet splitting plane

OSP: outlet splitting plane

U_m_: magnetically induced velocity

Fa: Fractional retrieval of samples at outlet a

Na: the number of particles at outlet *a*

Nb: the number of particles at outlet *b*

## Competing interests

The author(s) declare that they have no competing interests.

## Authors' contributions

HT: concept of the article and critical revision

YSF: supportive contributions

CBF: drafting of the manuscript and final approval
